# COVID-19, neurovascular thrombotic problem and short summary on blood coagulation disorder: a brief review

**DOI:** 10.1186/s41983-021-00442-w

**Published:** 2022-01-10

**Authors:** Rujittika Mungmunpuntipantip, Viroj Wiwanitkit

**Affiliations:** 1 Private Academic Consultant, Bangkok, Thailand; 2 Dr DY Patil University, Pune, India

**Keywords:** COVID-19, Neurovascular, Thrombosis, MeSH: thrombosis, Vein, Artery, Virus, Pathology

## Abstract

COVID-19 is the present global public health problem. This respiratory viral infection can manifest atypical presentation including neurological presentations. An important neurological problem in COVID-19 is neurovascular thrombosis. The basic pathogenesis of thrombosis in neurological system is explainable by the basic principle of thrombohemostasis. A hypercoagulability is a possible problem seen in some COVID-19 cases. In this brief review, the authors summarize venous and arterial thrombosis of neurovascular system as a complication of COVID-19. The updated pathophysiology of COVID-associated blood coagulation disorder is discussed. In addition, consideration regarding new COVID-19 vaccine related thrombotic adverse event is also raised.

## Introduction

Coronavirus disease 2019 (COVID-19) is the present global public health problem that has already caused pandemic since 2020. This respiratory coronaviral infection can cause severe respiratory illness and death might be the outcome in severe cases. COVID-19 can manifest several atypical clinical presentations including neurological presentation [1]. An important neurological problem is neurovascular thrombotic disorder [2–5]. A thrombotic event might be due to arterial or venous system affection.

The basic pathogenesis of thrombosis in neurological system is according to the basic principle of thrombohemostasis. A hypercoagulability is a possible problem observed in some COVID-19 cases. In a recent publication, Roushdy and Hamid mentioned for the disturbance in renin angiotensin system, angiotensin-converting enzyme (ACE) 2 receptors downregulation, endothelial cell damage, coagulopathy, cytokine storm, and platelet abnormality as underlying factors inducing abnormality in coagulation system [6]. The coagulation problem might affect several organs including neurological system [2–5]. In this brief review, the authors summarize on venous and arterial neurovascular system thrombotic disorder which is a possible complication of COVID-19. In addition, the updated pathophysiology of COVID-associated blood coagulation disorder is summarized and discussed. An additional consideration regarding new COVID-19 vaccine, the current primary prevention for COVID-19, related thrombotic adverse event is also raised.

## Reports on neurovascular thrombotic problem in COVID-19/COVID-19 vaccination

A neurovascular thrombotic problem is possible in any COVID-19 case. Sporadic case reports are published worldwide. The thrombohemostatic problem might occur at either venous or arterial system.

### Thrombosis in venous system

Sinus venous thrombosis a possible complication of COVID-19-associated hypercoagulability [1]. Zarrouk and Finsterer noted that sinus venous thrombosis was a complication of COVID-19- associated hypercoagulability [2]. Roushdy and Hamid mentioned that alteration of ACE 2 expression might result in hypoxemia, excessive blood pressure rise and volume overload that cause stroke [6].

Medicherla et al. noted that the problem could result in significant visual deficits and death [3]. Dakay et al. analyzed a case series of COVID-19 induced sinus venous thrombosis and noted that this thrombotic disorder should be suspected in any COVID patients with unexplained cerebral hemorrhage, or infarcts with an atypical pattern for arterial thrombotic disease [4]. Seizure might occur in some cases [5]. Hughes et al. mentioned that prophylaxis for thrombosis was required for any patients with COVID-19 [6]. For treatment, full dose anticoagulation is required and if there is a seizure, antiepileptic drug is indicated [5].

### Thrombosis in arterial system

According to a meta-analysis by Nannomi et al. [8], acute cerebrovascular diseases is not uncommon in patients with COVID-19. The problem might occur in a case with severe COVID-19 or pre-existing vascular problem [8]. According to a recent report from Milan, most COVID-19 cases with arterial thrombotic stroke occur within the first day of admission [9]. Problem is common in severe COVID-19 case [9]. However, cerebral arterial thrombosis in case with mild COVID-19 is also possible [10]. Large artery occlusion is possible [10]. An early thrombectomy might be useful but many cases still have sequelae after thrombectomy [10]. In an extreme rare case, both arterial and venous thrombotic disorders can concurrently occur [11].

Additional to thrombotic disorder due to COVID-19 illness, there are new reports on neurothrombotic disorder after COVID-19 vaccination. Since COVID19-vaccination is the best primary prevention against the new coronavirus infection, the neurological problem after vaccination is the current clinical concern. Similar to earlier discussion in COVID-19, there are sporadic reports on neurovascular thrombotic problem and COVID-19 vaccination. After vaccination, in some rare cases, the thrombohemostatic problem might occur. An incidence might occur at either venous or arterial system.

### Thrombosis in venous system

Vaccine-induced immune thrombotic thrombocytopenia and cerebral venous sinus thrombosis after COVID-19 vaccination is possible [12]. From a meta-analysis, the problem is usually seen after viral vector COVID-19 vaccine [12, 13]. In a recent report from Germany [13], an incidence of 0.02 to 0.15 per 100,000 person-months is reported and female has a more risk than male vaccine recipient. The common presenting symptom is severe headache [12]. Intracerebral hemorrhage and/or subarachnoid hemorrhage is observable in nearly half of the cases [12].

### Thrombosis in arterial system

Vaccine-induced immune thrombotic thrombocytopenia related acute ischemic stroke is possible [14]. Acute ischemic stroke after viral vector COVID-19 vaccination is sporadically reported [14]. The patient usually has a low platelet count [14]. Since thrombocytopenia is common, a mechanical thrombectomy is more preferable for treatment than intravenous thrombolysis [15]. Further studies to evaluate the outcome of different treatments and relationship with platelet/thrombohemostatic status is interesting.

An increased number of patients with neurovascular thrombosis when COVID-19 pandemic continues. Also, number of case reports of thrombosis after COVID-19 vaccine increases when there is a mass vaccination worldwide. Based on available data, it can confirm that there is clinical evidence to support possibility of thrombosis as complication in patient with COVID-19 or COVID-19 vaccine recipient. However, there is still no conclusive information regarding the exact pathogenesis. The pathogenesis should be complex and will be further discussed.

Also, conclusive recommendation for management of thrombosis is still not presently available. The management might be based on clinical and laboratory data on coagulation/thrombosis status on each individual patient.

## Pathophysiology of thrombotic disorder and problem in COVID-19/COVID-19 vaccination

Not only neurovascular thrombosis but also thrombosis at other organs are possible in COVID-19 patients/COVID-19 vaccine recipients. Additionally, some patients might have multiorgan thrombosis [16]. Therefore, it can confirm that the basic underlying pathophysiology should primarily involve the thrombohemostatic system. This is added to the well-established ideas that hyperinflammation and disruption of ACE2 system is the main cause of thrombosis [6]. That possible pathophysiology might explain problem in COVID-19 patient but might not in COVID-19 vaccine recipient. A Common pathophysiology for thrombosis based on abnormality of coagulation/thrombohemostatic system might give a good explanation. A basic pathophysiological process for thrombotic disorder is applied for neurovascular thrombosis due to COVID-19/COVID-19 vaccination.

### Thrombosis in venous system

Virchow triad is a basic principle that helps explain pathophysiology of COVID-19/COVID-19 vaccine induced neurovascular venous thrombosis. The three main components of the triad are hypercoagulability, endothelial injury and stasis [15]. Basic components that are associated with neurovascular thrombotic disorder in COVID-19/COVID-19 vaccination are abnormality of coagulation system, platelet and endothelium (Table 1). Both COVID-19 and COVID-19 vaccination can affect coagulation system and endothelium. In addition, the abnormality of platelet is a possible clinical problem resulted from COVID-19 or COVID-19 vaccination.

**Table 1:** Basic components stimulating neurovascular thrombotic disorder in COVID-19/COVID-19 vaccination

Components	COVID-19	COVID-19 vaccination
Platelet	Thrombocytosis due to hyperinflammtion process, thrombocytopenic thrombosis	Vaccine induced thrombocytopenia
Coagulation factor	Stimulation of coagulation cascade, DIC, SIC, microthrombi formation	Vaccine induced coagulopathy
Endothelium	Endothelium injury due to hyperinflammation process	Background vascular disorder
Blood viscosity	Infection induced hyperviscosity, previous COVID-19, background high blood viscosity status	Vaccine induce hyperviscosity, previous COVID-19, background high blood viscosity status
Static status	Prolonged hospitalization	Stasis in disable vaccine recipient

In COVID-19, hypercoagulability is a possible problem. Thrombocytosis is seen in COVID-19 [17, 18]. Coagulation cascade disturbance might be induced [18]. Various coagulopathies can occur. In COVID-19, activation of the coagulation cascade via tissue factor expression, and decreasing of fibrinolysis occur [18].

Disseminated intravascular coagulation (DIC), sepsis-induced coagulopathy (SIC) and local microthrombi formation can further result in venous thrombotic disorder [18].

Focusing on endothelial problem, the hyperinflammation occurs in COVID-19.

A host immune response can significantly contribute to vascular endothelial cell injury [17]. Finally, stasis also occurs in COVID-19. Infection can result in an increased blood viscosity and it can result in a stasis. According to a recent report, the higher increased blood viscosity occurs in case with COVID-19 reinfection [19].

For COVID-19 vaccination, the similar pathobiological process is possible. Vaccine might induce abnormal immune response and further cause problem. Vaccine related platelet disorder is sporadically reported [14, 15]. Additionally, the vaccination can result in an excessive blood viscosity [20]. A stasis can occur and result in thrombotic complication [20]. According to a recent report, the problem of hyperviscosity is higher after the second dose than the first dose of vaccine [20], hence, most neurovascular thrombotic event following COVID-19 vaccination usually follow the second dose of vaccination.

### Thrombosis in arterial system

In general, the neurovascular thrombosis usually occurs in a case with underlying vascular problem. A patient might have an underlying neurological disorder, such as cerebrovascular microinfarction, which could lead to a more complex clinical course when the patient gets COVID-19 illness [21]. Hence, a neurovascular arterial thrombosis is less common than venous thrombosis in COVID-19 [9]. As earlier mentioned, alteration of Virchow triad component occurs in COVID-19 and it might stimulate cerebral thrombotic stroke and infarction. In addition, another concurrent medical disorder, such as coinfection, can trigger neurovascular thrombotic disorders in patients with COVID-19 [21]. Nevertheless, the investigation for thrombosis should be performed for any COVID-19 case regardless history of underlying neurovascular problem [22].

Regarding COVID-19 vaccine related neurovascular thrombosis, the similar pathobiological process is possible. Vaccine might induce abnormal immune response and further cause problem [14, 15]. The underlying plays important role in determining occurrence of thrombotic event. As already noted, hyperviscosity and stasis might occur after COVID-19 vaccination. A patient with underlying neurological problem usually has a high blood viscosity, hence, that patient might have an increased risk of COVID-19 vaccination related neurovascular thrombosis. According to a recent study, the safety interval for thrombosis development is narrower in a vaccine recipient with underlying neurological disease [23]. It is recommended for a closed monitoring of possible thrombosis in a COVID-19 vaccine recipient who has an underlying neurological disease [23].

**Fig. 1:**
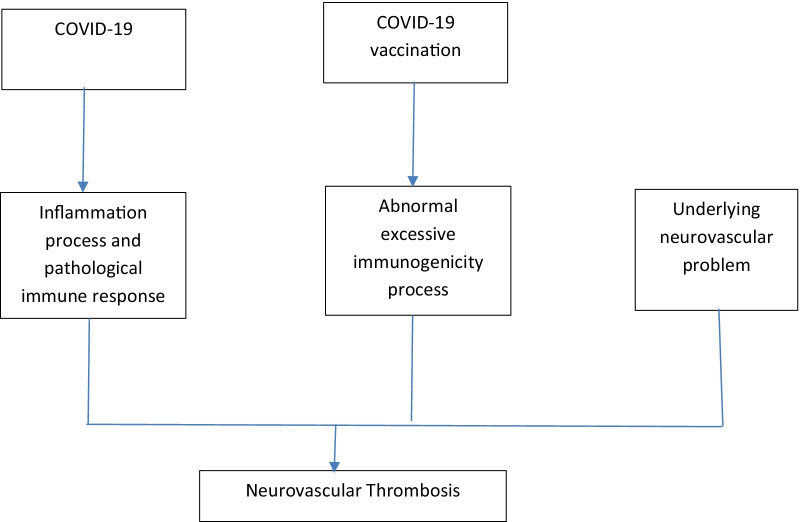
Pathophysiological process that might induce neurovascular thrombosis in COVID—19/COVID-19 vaccination

The authors hereby propose that the neurovascular thrombosis in COVID-19/COVID-19 vaccination is associated with disruption on basic hemostatic system (Fig. 1). Hyperviscosity is a main clinical problem that can cause thrombosis in COVID-19/COVID-19 vaccination. The change of blood viscosity occurs in COVID-19 and the pathogenesis might be due to the inflammation process and disruption of ACE2 system [7, 20]. For COVID-19 vaccination, the change of blood viscosity is not uncommon and the underlying pathogenesis is associated with hyperstimulation of immune response by COVID-19 vaccine that further cause an abrupt increased blood viscosity [21]. However, a remained problem for conclusion ion exact pathology is the confounding effect of underlying illness. Some patients might already have an underlying illness that can cause thrombosis regardless of COVID-19 or COVID-19 vaccination [24]. Some recent Arabic reports showed that the incidence of thrombocytopenia and peculiar thrombotic events might be more common in viral vector technology based COVID-19 vaccine [25, 26]. According to the recent management recommendation, mass COVID-19 vaccination should continue but with special caution [27].

## Future perspectives

As earlier mentioned, number of cases with COVID-19/COVID-19 vaccine related thrombosis should continue if the COVID-19 pandemic still persists. Although the main exact pathogenic mechanisms are unknown, both vaccine and host factors should play important roles [27]. More case reports and studies on clinical epidemiology, clinical feature and management of the cases will be the future trend. At present, the exact clinical interrelationship between COVID-19/COVID-19 vaccine r is still inconclusive. An important consideration is a data of background neurovascular status of the patient with thrombosis pre-COVID-19 or pre-vaccination is usually not available. Hence, it is still an inconclusive issue on effect of COVID-19/COVID-19 vaccination. The pathogenesis of COVID-19 and immunopharmacological reaction of COVID-19 vaccine are basically same but the clinical problem exists in some cases. Hence, it is a myth on the factors that can stimulate thrombosis problem. It is possible that a thrombosis in COVID-19 patient or COVID—19 vaccine recipient might be from unrecognized background neurovascular problem, thrombohemostatic disorder or conditions with high blood viscosity (such as metabolic syndrome [28, 29]). Therefore, further prospective studies on thrombosis incidence with a good investigation on background neurovascular condition of COVID-19 patient/COVID-19 vaccine recipient might provide a more conclusive data.

## Conclusions

A neurovascular thrombotic problem in COVID-19 is an important problem in clinical neurology. The hypercoagulability state might occur when there is a hyperinflammation caused by pathogenic virus. Increased blood cellular component and platelet might occur. A stimulation of coagulation cascade is also observed. Additionally, an increased plasma concentration or viscosity during COVID-19 illness is described. This pathophysiological process is observed in COVID-19 as well as COVID-19 vaccination. Rheological change of blood viscosity is another possible mechanism causing venous thrombosis following COVID-19 or COVID-19 vaccination.

## Data Availability

Not applicable.

## Abbreviations

ACE: Angiotensin-converting enzyme
COVID-19: Coronavirus disease 2019
DIC: Disseminated intravascular coagulation
SIC: Sepsis-induced coagulopathy
